# Analysis and simulation of a novel stochastic RNA silencing model

**DOI:** 10.1038/s41598-025-04142-w

**Published:** 2025-06-20

**Authors:** Ragaa Ahmed, Hillal M. Elshehabey

**Affiliations:** Department of Mathematics, Faculty of Science, South Valley University, Qena, 83523 Egypt

**Keywords:** RNA, Stochastic model, White noise, Existence and uniqueness, Lyapunov function, Applied mathematics, Computational science

## Abstract

The current work aims to investigate how random environmental fluctuations affect the dynamics of the RNA Silencing model. To capture this complexity, a novel stochastic RNA Silencing model is proposed by incorporating four distinct white noise terms into key system parameters. Unlike previous deterministic approaches, our model explicitly accounts for stochastic perturbations using Brownian motion processes. A comprehensive analysis of both the deterministic as well as the stochastic one are presented. Employing Lyapunov analysis, the stochastic system yields a unique global positive solution for any initial value, ensuring its biological relevance. Lastly, numerical simulations based on the Milstein’s higher-order-method are conducted for the two models. The findings highlight the significant influence of stochasticity on RNA silencing dynamics, offering new insights into the stability and behavior of gene regulatory processes under random fluctuations.

## Introduction

The RNA silencing is a normal biological mechanism, often referred to as RNA interference (RNAi), limits the action of particular genes to control gene expression. It is essential for controlling the expression of genes, protecting the body from viruses, preserving the integrity of the genome, and promoting the development of biotechnology^1–4^.

Double-stranded RNA (dsRNA) caused sequence-specific gene silencing in the nematode worm Caenorhabditis elegans, which is how the phenomenon of RNAi was originally discovered. Indeed, several significant participants are involved in the RNA silencing model, namely, (1) Small RNAs (brief RNA molecules): These usually consist of $20-30$ nucleotides (nucleotide is a compound consisting of a nucleoside linked to a phosphate group. Nucleotides form the basic structural unit of nucleic acids such as DNA.). Among these are small interfering RNAs (siRNAs) and microRNAs (miRNAs). (2) Dicer: Short double-stranded RNA (dsRNA) molecules are broken up into smaller RNA fragments by the enzyme Dicer, that is then added to the RNA-induced silencing complex (RISC). (3) RNA-induced silencing complex (RISC): It is a multiprotein complex that has a small RNA fragment and directs the complex to target RNAs. (4) Target RNA (the mRNA molecule): It is complementary to the small RNA fragment loaded onto the RISC. The binding of the RISC to the target RNA results in its degradation or translational repression, thereby silencing gene expression^5,6^.

Apart from experimental observation of such actual phenomena, alternative techniques can be used to control such mechanisms by mathematical models. Particularly, in the study of infectious diseases, mathematical models have proven to be a valuable tool. Further, studying their behaviors and how they spread becomes easier through the utilization of dynamical systems. Bergstrom et al.^2^established a series of mathematical models for RNA silencing. It was demonstrated that existing models provide limited insight into the mechanisms that prevent erroneous reactions from silencing essential genes in an organism. Hence, they extended the basic models to illustrate that the amplification process, previously assumed to be unidirectional, may have a more complex nature. An overview of the significance and applications of RNA interference (RNAi) in gene silencing was provided by Malakondaiah et al.^7^. Niu et al.^8^ reviewed recent advances in understanding the role of RNA interference (RNAi) in antiviral defense and its application as a pest control strategy in insects, highlighting gaps between fundamental RNAi mechanisms and their practical implementation in pest management. Review of challenges and future prospects of RNA based gene silencing modalities to control insect and fungal plant pests was provided in the work of Choudry et al.^9^. The review focuses on key delivery strategies of dsRNA molecules, comprising host-induced gene silencing, spray-induced gene silencing (SIGS), and virus-induced gene silencing, their strengths and weaknesses, and efficiency against phytophagous insects and fungal pathogens.

Further, numerous works regarding epidemics, including SIR, SEIR, RNA and others, are available^10–15^.

Whereas deterministic models are commonly utilized to examine the dynamics of pandemic propagation, environmental noise can affect these models. Conversely, stochastic differential equations provide a more accurate description such phenomena, especially in comprehending the dynamics of RNA silencing^16^. Indeed, as models are undoubtedly exposed to ambient white noise, it is crucial to disclose how the noise impacts the epidemic models. In biological and physical systems, the noise can have significant impacts. Thus, the existence of such noise source results in a behavior alteration of the corresponding deterministic development of the system^17^.

Numerous mathematical strategies, depends on interacting particle systems (IPS), have been developed during the last forty years in order to substitute stochastic dynamics for deterministic dynamics, with surprising results^18^. Hence, the derivation of stochastic partial differential equation (SPDEs) are established^19–22^. These type of differential equation i.e., SPDEs have a notable impact on various branches of applied scientific fields, such as disease dynamics^1,14,23–39^. Among these works, the work of Settati et al.^36^ related to the stochastic SIR epidemic model dynamics on scale-free networks, in which a stochastic SIR model on complex networks, using a scale-free network to simulate inter-human interactions is presented. Also, Lahrouz et al.^37^ expressed the impacts of randomness suppress backward bifurcation in an epidemic model with limited medical resources. This paper explores the dynamic characteristics of a stochastic SIR epidemic model with a transmission rate influenced by white noise perturbations. Stochastic SIRS epidemic model was proposed in^39^. This study presents a novel stochastic variation of the SIRS model, emphasizing perturbations in the immunity decay rate. The stochastic threshold of the COVID-19 epidemic model was presented in^38^. This study investigates the complexities of epidemic modeling in uncertain environments. By integrating white noise and Le’vy noise, it determines a comprehensive framework to capture the dynamic behavior of the COVID-19 epidemic incorporating jump perturbations.

Motivated by the above mentioned references, the main aim here is to present the mathematical investigation of stochastic RNA Silencing model as well as its deterministic counter part. To the best of the author’s knowledge, no prior study has studied the stochastic RNA Silencing model. Hence, the main objective of this work are to provide a comprehensive mathematical investigation of RNA Silencing model in both its stochastic and deterministic forms. This involves investigating the behavior, dynamics, and potential implications of the model using mathematical techniques. By analyzing the model from theses two perspectives, this study seeks to accumulate the understanding of RNA interference dynamics and contribute to the broader field of mathematical biology. The findings are expected to offer new insights into the role of stochasticity in gene regulation and provide a foundation for future research in this area. This paper is organized as follows: in Sect. 2, the RNA Silencing model with mathematical details is presented. Section 3 is devoted to the dynamics of the stochastic RNA silencing model. Numerical results are simulated in Sect. 4, followed by a conclusion remarks in Sect. 5.

## RNA silencing model

In the current section, we present the RNA silencing deterministic model. Also, establish in details its mathematical analysis, stability which was not found in the literature. The RNA silencing model, originally introduced by Bergstrom et al.^2^.

In this model, at time *t*, *S*(*t*) represents the quantity of the dsRNA, *R*(*t*) corresponds to the amount of RISC, *C*(*t*) expresses the magnitude of RISC-mRNA-complex, and *M*(*t*) states for the mRNA. Hence, we have the following differential equation system

1 $$\begin{aligned} \begin{aligned} \frac{dS(t)}{dt}&=-\iota _a S(t)+\iota _g C(t),\\ \frac{dR(t)}{dt}&=n \iota _a S(t)-\iota _{r}R(t)-\iota _b R(t)M(t),\\ \frac{dC(t)}{dt}&=\iota _b R(t)M(t)-(\iota _g+\iota _c)C(t),\\ \frac{dM(t)}{dt}&=\iota _h-\iota _{m}M(t)-\iota _b R(t)M(t). \end{aligned} \end{aligned}$$

In this model (1), all variables and parameters are assumed to be positive values. Further, their meaning and values are listed in Table 1.

**Table 1:** Meaning and values of the parameters in the system (1).

Symbol	Description	Value^2^
*n*	Amount of siRNAs generated from a single secondary dsRNA [for each molecule]	5
$\iota _a$	Rate of Dicer-measured dsRNA degradation [for each molecule/time unit]	10
$\iota _b$	Constant mass action rate for RISC mRNA formation [for each (molecule)$^2$ / time unit]	0.001
$\iota _{c}$	Rate in which a complex is collapsed [for each complex / unit time]	1
$\iota _h$	Rate of mRNA synthesis rate [for each cell / time unit]	1000
$\iota _g$	Rate at which the RISC-mRNA-complex synthesizes dsRNA [for each complex / time unit]	1
$\iota _{m}$	Degradation amount of non-specific mRNA [for each molecule / time unit]	1
$\iota _{r}$	The RISC degradation rate [for each RISC / time unit]	0.1
*S*(0)	Initial value of dsRNA	10 or 1000
*R*(0)	Initial value of RISC	0
*C*(0)	Initial value of complex	0
*M*(0)	Initial value of mRNA	1000

### Equilibrium points and reproduction number

In order to determine the steady state of system 1, one can put $\frac{dS}{dt}=0,\frac{dR}{dt}=0,\frac{dC}{dt}=0,$ and $\frac{dM}{dt}=0$, that is

2 $$\begin{aligned} \begin{aligned} 0&=-\iota _a S(t)+\iota _g C(t),\\ 0&=n \iota _a S(t)-\iota _{r}R(t)-\iota _b R(t)M(t),\\ 0&=\iota _b R(t)M(t)-(\iota _g+\iota _c)C(t),\\ 0&=\iota _h-\iota _{m}M(t)-\iota _b R(t)M(t). \end{aligned} \end{aligned}$$

Solving this system leads to two steady states as follows^2^:

- The first steady state is $\xi ^0=(0,0,0,\frac{\iota _h}{\iota _{m}})$ and the silencing does not occur.
- The second steady state is $\xi ^*=(S^*,R^*,C^*,M^*)$ where
  $$\begin{aligned} S^*=\frac{\iota _g}{\iota _a}C^*,\ R^*=\frac{\iota _g(n-1)-\iota _c}{\iota _r}C^*,M^*=\frac{(\iota _g+\iota _c)\iota _r}{\iota _b (\iota _g(n-1)-\iota _c)},C^*=\frac{\iota _h}{\iota _g+\iota _c}-\frac{\iota _m\iota _r}{\iota _g(n-1)-\iota _c}. \end{aligned}$$
  Only when $S^*, R^*, C^*$, and $M^*$ are non-negative this second state does have biological significance; in this case, $\iota _h (\iota _g(n-1)-\iota _c)>\iota _m\iota _c\iota _r$ is necessary and sufficient^2^.

Now, following^27,40,41^, the basic reproduction number can be computed for the RNA silencing model in 1 (deterministic system) based on the standard Next-Generation Matrix (NGM) approach. Hence, suppose

$$\begin{aligned} & f(\xi )= \begin{bmatrix} 0 \\ 0 \\ \iota _bRM \\ 0 \end{bmatrix} \\ & \quad v(\xi )= \begin{bmatrix} \iota _aS-\iota _gC \\ -\iota _anS+\iota _r+\iota _bRM \\ (\iota _g+\iota _c)C \\ -\iota _h+\iota _m M+\iota _bRM \end{bmatrix} \end{aligned}$$

with, $\xi =[S,R,C,M]^T$ be the state vector. Using the free equilibrium $\xi ^0$ for the model, the ODEs of the model in 1 can be rewritten as

3 $$\begin{aligned} \frac{d\xi }{dt}=f(\xi )-v(\xi ) \end{aligned}$$

where function $v(\xi )$ expresses the frequency of new infections developing in each compartment that corresponds to it, and the rate of all potential transitions between a specific compartment and all other infected compartments is function $f(\xi )$^27,42,43^. Hence, based on the NGM technique, we have

$$\begin{aligned} & \textrm{F}= \frac{\partial f_i(\xi )}{\partial \xi _j}\vert _{\xi ^0}= \begin{bmatrix} 0& 0& 0& 0 \\ 0& 0& 0& 0 \\ 0& \iota _bM& 0& \iota _bR \\ 0& 0& 0& 0 \end{bmatrix}_{\xi ^0}=\begin{bmatrix} 0& 0& 0& 0 \\ 0& 0& 0& 0 \\ 0& \frac{\iota _b\iota _h}{\iota _m}& 0& 0 \\ 0& 0& 0& 0 \end{bmatrix}, \\ & \quad \mathrm V= \frac{\partial v_i(\xi )}{\partial \xi _j}\vert _{\xi ^0}= \begin{bmatrix} \iota _a& 0& -\iota _g& 0 \\ -\iota _a n& \iota _r+\iota _b M& 0& \iota _bR \\ 0& 0& (\iota _g+\iota _c)& 0 \\ 0& \iota _bM& 0& \iota _m+\iota _bR \end{bmatrix}_{\xi ^0}=\begin{bmatrix} \iota _a& 0& -\iota _g& 0 \\ -\iota _a n& \iota _r+\frac{\iota _b \iota _h}{\iota _m}& 0& 0 \\ 0& 0& (\iota _g+\iota _c)& 0 \\ 0& \frac{\iota _b \iota _h}{\iota _m}& 0& \iota _m \end{bmatrix}, \end{aligned}$$

for all the components of *f* and $\xi$ i.e., $1\le i,j\le 4$. The NGM is computed by $FV^{-1}$ and, ${\mathcal {R}}_0 = \rho (FV^{-1})$, where $\rho$ is the spectral radius. Hence,

4 $$\begin{aligned} {\mathcal {R}}_0=\frac{n\iota _g\iota _b\iota _h}{(\iota _g+\iota _c)(\iota _b\iota _h+\iota _r \iota _m)}. \end{aligned}$$

### Existence and uniqueness

Here, we present the mathematical analysis for the existence of at most one solution to the system 1. For this, we follow the technique presented in^44,45^, thus, we start by rewrite system 1 as

5 $$\begin{aligned} \begin{aligned} G_S(t,\phi )=\frac{dS}{dt}&=-\iota _a S(t)+\iota _g C(t),\\ G_R(t,\phi )=\frac{dR}{dt}&=n \iota _a S(t)-\iota _{r}R(t)-\iota _b R(t)M(t),\\ G_C(t,\phi )= \frac{dC}{dt}&=\iota _b R(t)M(t)-(\iota _g+\iota _c)C(t),\\ G_M(t,\phi )=\frac{dM}{dt}&=\iota _h-\iota _{m}M(t)-\iota _b R(t)M(t). \end{aligned} \end{aligned}$$

with, $\phi =\xi ^T=(S,R,C,M)$. Now, let $t_f$ be the final time, $t\in [0,t_f]$ and assume that $G_S, G_R, G_C,$ and $G_M$ are bounded such that $||S||_\infty<N_S, ||R||_\infty<N_R,||C||_\infty <N_C$ and $||M||_\infty <N_M$. Hence, in order to complete our goal we have to show that $G_S, G_R, G_C,$ and $G_M$ remain linear growth, and also Lipschitz conditions holds correctly. To prove the linear growth property, we have for the first equation in the system 5:

6 $$\begin{aligned} \begin{aligned} \vert G_S(t,\phi )\vert&=\vert -\iota _aS(t)+\iota _gC(t)\vert , \\&\le \iota _a\vert S\vert +\iota _g\vert C\vert \\&< \iota _a\sup _{t\in D_{S}}\vert S\vert +\iota _g\sup _{t\in D_{C}}\vert C\vert \\&<\iota _aN_S+\iota _gN_C=N_{SS}<\infty . \end{aligned} \end{aligned}$$

Similarly, for the second equation of the system 5

7 $$\begin{aligned} \begin{aligned} \vert G_R(t,\phi )\vert&=\vert \iota _anS(t)-\iota _r R(t)-\iota _b R(t)M(t)\vert ,\\&\le \iota _a n\vert S\vert +\iota _r \vert R\vert +\iota _b\vert R\vert \vert M\vert \\&< \iota _a n\sup _{t\in D_{S}}\vert S\vert + \iota _r\sup _{t\in D_{R}}\vert R\vert +\iota _b \sup _{t\in D_{R}}\vert R\vert \sup _{t\in D_{M}}\vert M\vert \\&< \iota _a n N_S+\iota _r N_R+\iota _b N_{R}N_{M}=N_{RR}<\infty . \end{aligned} \end{aligned}$$

Also, for the third equation of the system 5

8 $$\begin{aligned} \begin{aligned} \vert G_C(t,\phi )\vert&=\vert \iota _b R(t)M(t)-(\iota _g+\iota _c)C(t)\vert \\&\le \iota _b\vert R\vert \vert M\vert +(\iota _g+\iota _c)\vert C\vert \\&< \iota _b\sup _{t\in D_{R}}\vert R\vert \sup _{t\in D_{M}}\vert M\vert +(\iota _g+\iota _c)\sup _{t\in D_{C}}\vert C\vert \\&< \iota _b N_RN_{M}+(\iota _g+\iota _c)N_C=N_{CC}<\infty . \end{aligned} \end{aligned}$$

Lastly, for the fourth equation of the system 5

9 $$\begin{aligned} \begin{aligned} \vert G_M(t,\phi )\vert&=\vert \iota _h-\iota _m M(t)-\iota _bR(t)M(t)\vert \\&\le \iota _h+ \iota _m \vert M\vert +\iota _b\vert R\vert \vert M\vert \\&< \iota _h+\iota _m\sup _{t\in D_{M}}\vert M\vert +\iota _b\sup _{t\in D_{R}}\vert R\vert \sup _{t\in D_{M}}\vert M\vert \\&< \iota _h+ \iota _m N_M+\iota _b N_{R}N_M= N_{MM}<\infty . \end{aligned} \end{aligned}$$

This concludes that the system’s growth is linear. Now, the proof of the Lipschitz conditions is as follows

10 $$\begin{aligned} \begin{aligned} \vert G_S(t,\phi _{S_{1}})-G_S(t,\phi _{S_{2}})\vert&=\vert -\iota _a (S_1-S_2)\vert ,\\&\le \iota _a\vert S_1-S_2\vert . \end{aligned} \end{aligned}$$

Also, for the second equation, we have

11 $$\begin{aligned} \begin{aligned} \vert G_R(t,\phi _{R_{1}})-G_R(t,\phi _{R_{2}})\vert&=\vert -(R_1-R_2)(\iota _r+\iota _bM)\vert ,\\&\le \vert (R_1-R_2)\vert (\iota _r+\iota _bN_M)\\&\le \kappa _R\vert R_1-R_2\vert , \end{aligned} \end{aligned}$$

where, $\kappa _R=(\iota _r+\iota _bN_M)$. Similarly, for the third equation, we have

12 $$\begin{aligned} \begin{aligned} \vert G_C(t,\phi _{C_{1}})-G_C(t,\phi _{C_{2}})\vert&=\vert -(\iota _g+\iota _c)(C_1-C_2)\vert \\&\le (\iota _g+\iota _c)\vert C_1-C_2\vert \\&\le \kappa _C\vert C_1-C_2\vert , \end{aligned} \end{aligned}$$

where, $\kappa _C=\iota _g+\iota _c$. Lastly, the fourth equation of this system gives

13 $$\begin{aligned} \begin{aligned} \vert G_M(t,\phi _{M_{1}})-G_M(t,\phi _{M_{2}})\vert&=\vert -(M_1-M_2)(\iota _m+\iota _b R)\vert \\&\le \vert (M_1-M_2)\vert (\iota _m+\iota _b N_M)\\&\le \kappa _M \vert (M_1-M_2)\vert , \end{aligned} \end{aligned}$$

where, $\kappa _M=\iota _m+\iota _b N_M$, and $\{\iota _a,\kappa _R,\kappa _C,\kappa _M\}$ are all positive constants. Thus, the linear growth and Lipschitz conditions are satisfied by each of the four functions $G_S,G_R,G_C$ and $G_M$ in the system 5. Hence, the model admits at most one solution.

## Dynamics of stochastic RNA silencing model

We start first by some relevant concepts from the mathematical probability theory and stochastic differential equations that are needed later in the analysis of the work^46–48^.

### Definition 1

A probability space is defined as the triple $(\Omega , {\mathcal {F}},{\mathbb {P}})$, with $\Omega$ be a set, ${\mathcal {F}}$ be a $\sigma$-algebra of subsets of $\Omega$, and ${\mathbb {P}}$ be a mapping from ${\mathcal {F}}$ into [0, 1] satisfying ${\mathbb {P}}(\emptyset )=0$, ${\mathbb {P}}(\Omega )=1$ and ${\mathbb {P}}(\bigcup _{i=1}^{\infty }A_i)=\sum _{i=1}^{\infty }{\mathbb {P}}(A_i)$, with $A_i\bigcap A_j=\emptyset$ holds for all $i\ne j$.

### Definition 2

A mapping $X:\Omega ; {\mathbb {R}}$ satisfying $\{\omega \vert X(\omega )\le t\}\in {\mathcal {F}} \ \forall t \in {\mathbb {R}}$ defines random variable *X*.

### Definition 3

The expected value of a random variable *X*, defined on some probability space $(\Omega , {\mathcal {F}},{\mathbb {P}})$ is defined as

$$\begin{aligned} {\mathbb {E}}[X]:=\int _{\Omega }X d{\mathbb {P}}, \end{aligned}$$

where, its variance is given by

$$\begin{aligned} Var(X)={\mathbb {E}}[(X-{\mathbb {E}}(X))^2]={\mathbb {E}}[X^2]-({\mathbb {E}}[X])^2. \end{aligned}$$

### Definition 4

A random variable *X* is called normal, or Gaussian $N(\mu , \lambda ^2)$ with mean $\mu$, and variance $\lambda ^2$ if $\forall$ $-\infty \le a\le b\le \infty$

$$\begin{aligned} {\mathbb {P}}(a\le X\le b)=\frac{1}{\sqrt{2\pi \lambda ^2}}\int _{a}^{b}e^{-\frac{(x-\mu )^2}{2\lambda ^2}}dx. \end{aligned}$$

### Definition 5

A set of random variables $X(t) (0 \le t < \infty )$, each established on the same probability space $(\Omega ,{\mathcal {F}},{\mathbb {P}}),$ construct a stochastic process. The $\omega$-th sample route of the process is represented by the mapping $t\mapsto X(t, \omega )$.

### Definition 6

A Brownian motion, or a standard Wiener process, over [0, *T*] is a stochastic process ${\mathcal {B}}(t)$ for $t\in [0,T]$ whenever it satisfies the following three conditions

- $${\mathcal {B}}(0)=0$$,
- the random variable given by the increment ${\mathcal {B}}(t)-{\mathcal {B}}(s)$ is $N(0,t-s)$; equivalently, ${\mathcal {B}}(t)-{\mathcal {B}}(s)~\sqrt{t-s} N(0,1)$ for $0\le s\le t\le T$,
- For $0\le s\le t\le u\le v \le T$, the difference ${\mathcal {B}}(t)-{\mathcal {B}}(s)$ and ${\mathcal {B}}(v)-{\mathcal {B}}(u)$ are autonomous (not dependent).

Now, we define a *d*-dimensional stochastic differential equation (SDE) for $t\ge t_0$ with initial data $x(t_0)=x_0\in {\mathbb {R}}^d$ in general as

14 $$\begin{aligned} dx(t)=f(x(t),t)dt+g(x(t),t)d{\mathcal {B}}(t), \end{aligned}$$

Associated with this equation the differential operator ${\mathcal {L}}$ given as

15 $$\begin{aligned} {\mathcal {L}}=\frac{\partial }{\partial t}+\sum _{i=1}^{d}f_i(x,t)\frac{\partial }{\partial x_i}+\frac{1}{2}\sum _{i,j=1}^{d}[g^T(x,t)g(x,t)]_{ij}\frac{\partial ^2}{\partial x_i\partial x_j}. \end{aligned}$$

### The stochastic model

As mentioned above, the fundamental aim of the current work is to incorporate the impact of environmental noise into system 1. Hence, the fluctuations of the four parameters $\iota _a,\iota _r,\iota _c$ and $\iota _m$ are assumed random as follows

16 $$\begin{aligned} \begin{aligned} \iota _a\rightarrow&\iota _a + \sigma _1 \ d{\mathcal {B}}_1(t)\\ \iota _r\rightarrow&\iota _r+ \sigma _2 \ d{\mathcal {B}}_2(t)\\ \iota _c\rightarrow&\iota _c + \sigma _3 \ d{\mathcal {B}}_3(t)\\ \iota _m\rightarrow&\iota _m+ \sigma _4 \ d{\mathcal {B}}_4(t) \end{aligned} \end{aligned}$$

with $\sigma _i^2>0, i=1,\cdots , 4$ are the intensity of environmental white noise. It is worth to mention here that in the real world, because of ecological fluctuations, each of the parameters associated with the deterministic system displays random fluctuations to varying degrees, not only $\iota _a,\iota _r,\iota _c$, and $\iota _m$ that are influenced by random noise. Under this assumption, the model 1 turns into

17 $$\begin{aligned} \begin{aligned} dS(t)&=\Big (-\iota _a S(t)+\iota _g C(t)\Big )dt-\sigma _1 S(t) d{\mathcal {B}}_1(t),\\ dR(t)&=\Big (\iota _anS(t)-\iota _r R(t)-\iota _b R(t)M(t)\Big )dt+\sigma _1 n S(t) d{\mathcal {B}}_1(t)-\sigma _2R(t)d{\mathcal {B}}_2(t)\\ dC(t)&=\Big (\iota _b R(t)M(t)-(\iota _g+\iota _c)C(t)\Big )dt-\sigma _3 C(t)d{\mathcal {B}}_3(t), \\ dM(t)&=\Big (\iota _h-\iota _m M(t)-\iota _b R(t)M(t)\Big )dt-\sigma _4 M(t)d{\mathcal {B}}_4(t). \end{aligned} \end{aligned}$$

If $\sigma _1=\sigma _2=\sigma _3=\sigma _4=0$, then we have the deterministic counterpart of the above model that is model 1. In order to examine this stochastic model, we first use the Lyapunov analysis approach described in^49^ to demonstrate the existence of at most one global positive solution for this system. This result is concluded in the following theorem assuming ${\mathbb {R}}^4_{+}:=\{(S,R,C,M)\in {\mathbb {R}}^4; S,R,C,M>0\}$.

#### Theorem 1

The system 17 admits at most one solution $(S(t),R(t),C(t),M(t))\in {\mathbb {R}}^4$ for given initial value $(S(0), R(0), C(0),M(0))\in {\mathbb {R}}^4_{+}$ and every $t\ge 0$. Also, this solution remains positive with probability one.

#### Proof

Following the work of Mao et al.^47,50^ and its application for a AIDS system in^49^, the proof of the theorem can be concluded as follows:

Firstly, the system 17 with the given initial value $(S(0), R(0), C(0), M(0))\in {\mathbb {R}}^4_{+}$ has at most one local solution (*S*(*t*), *R*(*t*), *C*(*t*), *M*(*t*)), on $[0, {\mathfrak {t}}_e )$, since its coefficients are locally Lipschitz continuous. Here, ${\mathfrak {t}}_e$, is the explosion time which is well defined in the^47,50^.

Secondly, showing the existence of unique global solution that is to show that ${\mathfrak {t}}_e=\infty$ a.s. (almost surely). Suppose ${\mathfrak {L}}_0>0$ be sufficiently large enough that the initial conditions *S*(0), *R*(0), *C*(0), *M*(0) are in the interval $[\frac{1}{{\mathfrak {L}}_0},{\mathfrak {L}}_0]$. Define for each integer ${\mathfrak {L}}\ge {\mathfrak {L}}_0$ the stopping time as

18 $$\begin{aligned} {\mathfrak {t}}_{\mathfrak {L}}=\inf \{t\in [0;{\mathfrak {t}}_e):\min \{S(t),R(t),C(t),M(t)\}\le \frac{1}{{\mathfrak {L}}} \quad \text {or}\quad \max \{S(t),R(t),C(t),M(t)\}\ge {\mathfrak {L}}\}, \end{aligned}$$

which is rising as ${\mathfrak {L}}\rightarrow \infty$ i.e. ${\mathfrak {t}}_\infty =\lim _{{\mathfrak {L}}\rightarrow \infty }{\mathfrak {t}}_{\mathfrak {L}}$. It obvious that ${\mathfrak {t}}_\infty \le {\mathfrak {t}}_e$ a.s. Hence, in order to complete the proof, we have only to prove that ${\mathfrak {t}}_\infty =\infty$ a.s., then ${\mathfrak {t}}_e=\infty$ and $(S(t),R(t),C(t),M(t))\in {\mathbb {R}}^4_{+}$ a.s. for all $t\ge 0$. We prove this by contradiction. To this end, assume ${\mathfrak {t}}_\infty =\infty$ a.s. is not true, then one can find constants $T>0$ and $\varepsilon \in (0,1)$ satisfying ${\mathbb {P}}\{{\mathfrak {t}}_{\mathfrak {L}}\le T\}>\varepsilon$. Thus, an integer ${\mathfrak {L}}_1\ge {\mathfrak {L}}_0$ exists such that

19 $$\begin{aligned} {\mathbb {P}}({\mathfrak {t}}_{\mathfrak {L}}\le T)\ge \varepsilon \quad \text {for all}\quad {\mathfrak {L}}\ge {\mathfrak {L}}_1. \end{aligned}$$

Define the function ${\mathcal {V}} : {\mathbb {R}} _+ ^ 4 \rightarrow {\mathbb {R}} _+$ being non-negative (${\mathcal {V}}\ge 0$) and twice differentiable (${\mathcal {V}}\in {\mathbb {C}} ^2$) given by the following form:

20 $$\begin{aligned} \begin{aligned} {\mathcal {V}}(S,R,C,M)=&(S-1-\log S)+(R-1-\log R)+(C-1-\log C)\\&+(M-1-\log M). \end{aligned} \end{aligned}$$

Based on It${\hat{o}}$’s formula^51^, we have

21 $$\begin{aligned} \begin{aligned} d{\mathcal {V}}=&\Big (1-\frac{1}{S}\Big )\Bigg (\Big (-\iota _a S(t)+\iota _g C(t)\Big )dt-\sigma _1 S(t)d{\mathcal {B}}_1(t)\Bigg )\\&+\frac{1}{2S^2}\Bigg (\Big (-\iota _a S(t)+\iota _g C(t)\Big )dt-\sigma _1 S(t) d{\mathcal {B}}_1(t)\Bigg )^2\\&+(1-\frac{1}{R})\Bigg (\Big (\iota _a nS(t)-\iota _r R(t)-\iota _b R(t)M(t)\Big )dt+\sigma _1 n S(t) d{\mathcal {B}}_1(t)-\sigma _2R(t)d{\mathcal {B}}_2(t)\Bigg )\\&+\frac{1}{2R^2}\Bigg (\Big (\iota _anS(t)-\iota _r R(t)-\iota _b R(t)M(t)\Big )dt+\sigma _1 n S(t) d{\mathcal {B}}_1(t)-\sigma _2R(t)d{\mathcal {B}}_2(t)\Bigg )^2\\&+(1-\frac{1}{C})\Bigg (\Big (\iota _b R(t)M(t)-(\iota _g+\iota _c)C(t)\Big )dt-\sigma _3 C(t)d{\mathcal {B}}_3(t)\Bigg )\\&+\frac{1}{2C^2}\Bigg (\Big (b R(t)M(t)-(\iota _g+\iota _c)C(t)\Big )dt-\sigma _3 C(t)d{\mathcal {B}}_3(t)\Bigg )^2\\&+(1-\frac{1}{M})\Bigg (\Big (\iota _h-\iota _m M(t)-\iota _b R(t)M(t)\Big )dt-\sigma _4 M(t)d{\mathcal {B}}_4(t)\Bigg )\\&+\frac{1}{2M^2}\Bigg (\Big (\iota _h-\iota _m M(t)-\iota _b R(t)M(t)\Big )dt-\sigma _4 M(t)d{\mathcal {B}}_4(t)\Bigg )^2, \end{aligned} \end{aligned}$$

then, with simple computations and putting $d{\mathcal {B}}(t)\cdot d{\mathcal {B}}(t)=d^2{\mathcal {B}}(t)=dt, dt\cdot d{\mathcal {B}}(t)=0, dt \cdot dt=0, d{\mathcal {B}}_1(t)\cdot d{\mathcal {B}}_2(t)=0$^52^, we have

22 $$\begin{aligned} \begin{aligned} d{\mathcal {V}}&=\Big (1-\frac{1}{S}\Big )\Bigg (\Big (-\iota _a S(t)+\iota _g C(t)\Big )dt-\sigma _1 S(t)d{\mathcal {B}}_1(t)\Bigg )\\&+(1-\frac{1}{R})\Bigg (\Big (\iota _anS(t)-\iota _r R(t)-\iota _b R(t)M(t)\Big )dt+\sigma _1 n S(t) d{\mathcal {B}}_1(t)-\sigma _2R(t)d{\mathcal {B}}_2(t)\Bigg )\\&+(1-\frac{1}{C})\Bigg (\Big (\iota _b R(t)M(t)-(\iota _g+\iota _c)C(t)\Big )dt-\sigma _3 C(t)d{\mathcal {B}}_3(t)\Bigg )\\&+(1-\frac{1}{M})\Bigg (\Big (\iota _h-\iota _m M(t)-\iota _b R(t)M(t)\Big )dt-\sigma _4 M(t)d{\mathcal {B}}_4(t)\Bigg )\\&+\frac{1}{2}\Big (\sigma _1^2+\sigma _2^2+\sigma _3^2+\sigma _4^2+\frac{\sigma _1^2n^2S^2}{R^2}\Big )dt, \end{aligned} \end{aligned}$$

that can be rewritten as

23 $$\begin{aligned} \begin{aligned} d{\mathcal {V}}&={\mathcal {L}} {\mathcal {V}} dt+\Big (R(1-S)+(R-1)nS\Big )\frac{\sigma _1}{R}\ d{\mathcal {B}}_1(t)+(1-R)\sigma _2d{\mathcal {B}}_2(t)\\&+(1-C)\sigma _3d{\mathcal {B}}_3(t)+(1-M)\sigma _4d{\mathcal {B}}_4(t), \end{aligned} \end{aligned}$$

in which the operator ${\mathcal {L}} {\mathcal {V}}:{\mathbb {R}}^4_{+}\rightarrow {\mathbb {R}}_{+}$ acts as

24 $$\begin{aligned} \begin{aligned} {\mathcal {L}} {\mathcal {V}}&=\frac{1}{2}\Big (\sigma _1^2+\sigma _2^2+\sigma _3^2+\sigma _4^2+\frac{\sigma _1^2n^2S^2}{R^2}\Big ) +\iota _a+\iota _anS+\iota _r+\iota _ M+\iota _g+\iota _c+\iota _h+\iota _m+\iota _bR\\&-\iota _as-\iota _c C-\iota _m M-\iota _b R M-\iota _r R-\frac{\iota _gC}{S}-\frac{\iota _a n S}{R}-\frac{\iota _b RM}{C}-\frac{\iota _h}{M}\\&\le \frac{\sigma _1^2}{2}+\frac{\sigma _4^2}{2}+\frac{\sigma _3^2}{2}+\frac{\sigma _2^2}{2}+\frac{\sigma _1^2n^2S^2}{2R^2}+\iota _a+\iota _anS+\iota _r+\iota _b M+\iota _g+\iota _c+\iota _h+\iota _m+\iota _bR\\ &:={\mathbb {F}}(S(t),M(t),R(t)), \end{aligned} \end{aligned}$$

and is bounded^53^. Performing the integrating of (23) from 0 to $\min (T,{\mathfrak {t}}_{{\mathfrak {L}}}):=T\wedge {\mathfrak {t}}_{{\mathfrak {L}}}$ and keeping in mind (24), then taking the mathematical expectation of the resulting equation, leads to

25 $$\begin{aligned} \begin{aligned} {\mathbb {E}}[ {\mathcal {V}}(S(T\wedge {\mathfrak {t}}_{{\mathfrak {L}}}),R(T\wedge {\mathfrak {t}}_{{\mathfrak {L}}}),C(T\wedge {\mathfrak {t}}_{{\mathfrak {L}}}),M(T\wedge {\mathfrak {t}}_{{\mathfrak {L}}}))]&\le {\mathcal {V}}\big (S(0),R(0),C(0),M(0)\big )\\&+{\mathbb {E}}\bigg [\int _0^{T\wedge {\mathfrak {t}}_{{\mathfrak {L}}}}{\mathbb {F}}(S(\zeta ),M(\zeta ),R(\zeta ))d\zeta \bigg ]\\ &+{\mathbb {E}}\bigg [\int _0^{T\wedge {\mathfrak {t}}_{{\mathfrak {L}}}}\Big (R(1-S)+(R-1)nS\Big )\frac{\sigma _1}{R}\ d{\mathcal {B}}_1(s)\bigg ]\\&+{\mathbb {E}}\bigg [\int _0^{t}(1-R)\sigma _2d{\mathcal {B}}_2(s)\bigg ]\\ &+{\mathbb {E}}\bigg [\int _0^{T\wedge {\mathfrak {t}}_{{\mathfrak {L}}}}(1-C)\sigma _3d{\mathcal {B}}_3(s)\bigg ]\\ &+{\mathbb {E}}\bigg [\int _0^{T\wedge {\mathfrak {t}}_{{\mathfrak {L}}}}(1-M)\sigma _4d{\mathcal {B}}_4(s)\bigg ]. \end{aligned} \end{aligned}$$

The last four terms in the right hand side of equation (25) are the quadratic variation of the stochastic integral and all are vanish^54,55^.

Then, we have

26 $$\begin{aligned} \begin{aligned}&{\mathbb {E}}[ {\mathcal {V}}(S(T\wedge {\mathfrak {t}}_{{\mathfrak {L}}}),R(T\wedge {\mathfrak {t}}_{{\mathfrak {L}}}),C(T\wedge {\mathfrak {t}}_{{\mathfrak {L}}}),M(T\wedge {\mathfrak {t}}_{{\mathfrak {L}}}))]\\&\le {\mathcal {V}}\big (S(0),R(0),C(0),M(0)\big )+{\mathbb {E}}\bigg [\int _0^{T\wedge {\mathfrak {t}}_{{\mathfrak {L}}}}{\mathbb {F}}(S(\zeta ),M(\zeta ),R(\zeta ))d\zeta \bigg ]. \end{aligned} \end{aligned}$$

Based on ${\mathbb {E}}[ {\mathcal {V}}(S(T\wedge {\mathfrak {t}}_{{\mathfrak {L}}}),R(T\wedge {\mathfrak {t}}_{{\mathfrak {L}}}),C(T\wedge {\mathfrak {t}}_{{\mathfrak {L}}}),M(T\wedge {\mathfrak {t}}_{{\mathfrak {L}}}))]>0$, and the definition of characteristic or indicator function ${\mathbb {I}}_{\Omega _{\mathfrak {L}}}$ of the set $\Omega _{\mathfrak {L}}:=\{{\mathfrak {t}}_{\mathfrak {L}}\le T\}$ for all ${\mathfrak {L}}_1 \le {\mathfrak {L}}$, then ${\mathbb {E}}[{\mathcal {V}}((\cdot )]$ can be decomposed into

27 $$\begin{aligned} \begin{aligned} {\mathbb {E}}[ {\mathcal {V}} (S(T\wedge {\mathfrak {t}}_{{\mathfrak {L}}}),R(T\wedge {\mathfrak {t}}_{{\mathfrak {L}}}),&C(T\wedge {\mathfrak {t}}_{{\mathfrak {L}}}),M(T\wedge {\mathfrak {t}}_{{\mathfrak {L}}}))]\\ &={\mathbb {E}}\bigg [ {\mathcal {V}}\Big (S(T\wedge {\mathfrak {t}}_{{\mathfrak {L}}}),R(T\wedge {\mathfrak {t}}_{{\mathfrak {L}}}),C(T\wedge {\mathfrak {t}}_{{\mathfrak {L}}}),M(T\wedge {\mathfrak {t}}_{{\mathfrak {L}}})\Big ){\mathbb {I}}_{\Omega _{\mathfrak {L}}}\bigg ]\\&\quad +{\mathbb {E}}\bigg [ {\mathcal {V}}\Big (S(T\wedge {\mathfrak {t}}_{{\mathfrak {L}}}),R(T\wedge {\mathfrak {t}}_{{\mathfrak {L}}}),C(T\wedge {\mathfrak {t}}_{{\mathfrak {L}}}),M(T\wedge {\mathfrak {t}}_{{\mathfrak {L}}})\Big ){\mathbb {I}}_{{\mathfrak {t}}_{{\mathfrak {L}}}> T}\bigg ]\\&\ge {\mathbb {E}}\bigg [ {\mathcal {V}}\Big (S(T\wedge {\mathfrak {t}}_{{\mathfrak {L}}}),R(T\wedge {\mathfrak {t}}_{{\mathfrak {L}}}),C(T\wedge {\mathfrak {t}}_{{\mathfrak {L}}}),M(T\wedge {\mathfrak {t}}_{{\mathfrak {L}}})\Big ){\mathbb {I}}_{\Omega _{\mathfrak {L}}}\bigg ], \end{aligned} \end{aligned}$$

by which equation (26) reads

28 $$\begin{aligned} \begin{aligned} {\mathbb {E}}\bigg [ {\mathcal {V}}\Big (S(T\wedge {\mathfrak {t}}_{{\mathfrak {L}}}),R(T\wedge {\mathfrak {t}}_{{\mathfrak {L}}}),C(T\wedge {\mathfrak {t}}_{{\mathfrak {L}}}),M(T\wedge {\mathfrak {t}}_{{\mathfrak {L}}})\Big ) {\mathbb {I}}_{\Omega _{\mathfrak {L}}}\bigg ]&\le {\mathcal {V}}\big (S(0),R(0),C(0),M(0)\big )\\&+{\mathbb {E}}\bigg [\int _0^{T\wedge {\mathfrak {t}}_{{\mathfrak {L}}}}{\mathbb {F}}(S(\zeta ),M(\zeta ),R(\zeta ))d\zeta \bigg ]. \end{aligned} \end{aligned}$$

Also, for every $\nu \in \Omega _{\mathfrak {L}}$, there exists at least one of the variables $S({\mathfrak {t}}_{{\mathfrak {L}}}), R({\mathfrak {t}}_{{\mathfrak {L}}}), C({\mathfrak {t}}_{{\mathfrak {L}}}), M({\mathfrak {t}}_{{\mathfrak {L}}})$ that equals $\frac{1}{{{\mathfrak {L}}}}$ or ${\mathfrak {L}}$. Hence ${\mathcal {V}}(S({\mathfrak {t}}_{{\mathfrak {L}}}), R({\mathfrak {t}}_{{\mathfrak {L}}}), C({\mathfrak {t}}_{{\mathfrak {L}}}), M({\mathfrak {t}}_{{\mathfrak {L}}}))$ is not less than ${\mathfrak {L}}-\log {\mathfrak {L}}-1$ or $\log {\mathfrak {L}}-1+\frac{1}{{\mathfrak {L}}}$, i.e.,

29 $$\begin{aligned} {\mathcal {V}}(S({\mathfrak {t}}_{{\mathfrak {L}}}), R({\mathfrak {t}}_{{\mathfrak {L}}}), C({\mathfrak {t}}_{{\mathfrak {L}}}), M({\mathfrak {t}}_{{\mathfrak {L}}}))\ge ({\mathfrak {L}}-\log {\mathfrak {L}}-1)\wedge (\log {\mathfrak {L}}-1+\frac{1}{{\mathfrak {L}}}). \end{aligned}$$

Therefore,

30 $$\begin{aligned} {\mathcal {V}}\big (S(0),R(0),C(0),M(0)\big )+{\mathbb {E}}\bigg [\int _0^{T\wedge {\mathfrak {t}}_{{\mathfrak {L}}}}{\mathbb {F}}(S(\zeta ),M(\zeta ),R(\zeta ))d\zeta \bigg ]\ge \bigg (({\mathfrak {L}}-\log {\mathfrak {L}}-1)\wedge (\log {\mathfrak {L}}-1+\frac{1}{{\mathfrak {L}}}) \bigg ){\mathbb {E}}({\mathbb {I}}_{\Omega _{\mathfrak {L}}}) \end{aligned}$$

that is

31 $$\begin{aligned} {\mathbb {P}} (\Omega _{\mathfrak {L}})\le \frac{V\big (S(0),R(0),C(0),M(0)\big )+{\mathbb {E}}\bigg [\int _0^{T\wedge {\mathfrak {t}}_{{\mathfrak {L}}}}{\mathbb {F}}(S(\zeta ),M(\zeta ),R(\zeta ))d\zeta \bigg ]}{\bigg (({\mathfrak {L}}-\log {\mathfrak {L}}-1)\wedge (\log {\mathfrak {L}}-1+\frac{1}{{\mathfrak {L}}})\bigg )} \end{aligned}$$

when ${\mathfrak {L}}\rightarrow \infty$, we get

32 $$\begin{aligned} {\mathbb {P}} (\Omega _\infty )={\mathbb {P}} ({\mathfrak {t}}_\infty \le T)=0 \end{aligned}$$

which is a contradiction with assumption (19). Hence it is not correct and ${\mathfrak {t}}_\infty =\infty$ a.s. Hence, the stochastic model has a unique global solution (*S*(*t*), *R*(*t*), *C*(*t*), *M*(*t*)) a.s.

$$\square$$

### Exponentially stability

We investigate here the stability of disease-free equilibrium for the stochastic system (17) stated in the following theorem.

#### Theorem 2

For $p \ge 2$ , the disease-free equilibrium point $\xi ^0=(0,0,0,\frac{\iota _{h}}{\iota _{m}}) \in {\mathbb {R}}^4_+$ of the system (17) is exponentially *p* -stable, if

33 $$\begin{aligned} \begin{aligned} \frac{\sigma _1^2(p-1)}{2}&<\iota _a\\ \frac{\sigma _2^2(p-1)}{2}&<\iota _{r}+\frac{\iota _{b}\iota _{h}}{\iota _m}\\ \frac{\sigma _3^2(p-1)}{2}&<\iota _g+\iota _c. \end{aligned} \end{aligned}$$

#### Proof

Assuming a Lyapunov function, with $p\ge 0$, given as

34 $$\begin{aligned} V=\frac{1}{p}\big (S^p+R^p+C^p+(\frac{\iota _{h}}{\iota _m}-M)^p\big ). \end{aligned}$$

Based on Ito’s formula, one can compute *dV*, from which ${\mathcal {L}}V$ can be concluded as

35 $$\begin{aligned} \begin{aligned} {\mathcal {L}}V=&-\bigg (\iota _m(\frac{\iota _h}{\iota _m}-M)^{p}+S^{p}\Big (a-0.5(p-1)\sigma _1^2\Big )+R^{p} \Big (\iota _{r}+\frac{\iota _b\iota _h}{\iota _m}-0.5(p-1)\sigma _2^2\Big )\\&+C^{p}\Big (\iota _g+\iota _c-0.5(p-1)\sigma _3^2\Big )\bigg )+R^{p-1}\iota _a n S(t)+0.5(p-1)R^{p-2}\sigma _1^2n^2S^2\\&+C^{p-1}R\frac{\iota _b\iota _h}{\iota _m}+\iota _gS^{p-1}C+\frac{\iota _b\iota _h}{\iota _m}R(t)(\frac{\iota _h}{\iota _m}-M)^{p-1}+0.5\sigma _4^2(p-1)(\frac{\iota _h}{\iota _m}-M)^{p-2}M^2. \end{aligned} \end{aligned}$$

In which all the terms with power $p-1$ and $p-2$ can be bounded using Lemma 1 in^56^ and we have

36 $$\begin{aligned} \begin{aligned} {\mathcal {L}}V\le&-\bigg (\iota _{m}(\frac{\iota _h}{\iota _m}-M)^{p}+\iota _aS^{p}+R^{p}\Big (\iota _{r}+\frac{\iota _b \iota _h}{\iota _{m}}\Big )+C^{p}\Big (\iota _g+\iota _c\Big )\bigg )\\&+\bigg (\frac{\iota _b\iota _h(p-1)\varepsilon }{p\iota _m}+\frac{0.5\sigma _4^2(p-1)(p-2)\varepsilon }{p}\bigg )(\frac{\iota _h}{\iota _m}-M)^{p}\\&+\bigg (\frac{2\iota _an\varepsilon ^{1-p}}{p}+\frac{(p-1)\sigma _1^2n^2\varepsilon ^{\frac{2-p}{p}}}{p} +\frac{\iota _g(p-1)\varepsilon }{p}+0.5(p-1)\sigma _1^2\bigg )S^p\\&+\bigg (\frac{\iota _an(p-1)\varepsilon }{p}+\frac{0.5(p-1)(p-2)\sigma _1^2n^2\varepsilon }{p}+\frac{4\iota _b \iota _h\varepsilon }{p\iota _{m}}+0.5(p-1)\sigma _2^2\bigg )R^p\\&+\bigg (\frac{\iota _b\iota _h(p-1)\varepsilon }{p\iota _{m}}+\frac{2\iota _g\varepsilon ^{1-p}}{p}+0.5(p-1)\sigma _3^2\bigg )C^p. \end{aligned} \end{aligned}$$

Then for $\varepsilon$ sufficiently small^56^, and assumption in Theorem 2, one can conclude that this system is exponentially *p* -stable. If $p=2$ then

37 $$\begin{aligned} \begin{aligned} \frac{\sigma _1^2}{2}&<\iota _a\\ \frac{\sigma _2^2}{2}&<\iota _{r}+\frac{\iota _{b}\iota _{h}}{\iota _m}\\ \frac{\sigma _3^2}{2}&<\iota _g+\iota _c, \end{aligned} \end{aligned}$$

and the equilibrium point $\xi ^0$ of system (17) will be globally asymptotically stable. $\square$

## Numerical scheme and simulations

We present here an accurate higher order numerical scheme for the suggested stochastic epidemic model (17) that is the Milstein’s higher order method^57^. The Milstein’s method is an extension of the well-known Euler-Maruyama method, incorporating additional terms to improve accuracy. That is an It$\hat{o}$ correction term, which accounts for nonlinear interactions of the noise process, resulting in a converging rate of $O(\Delta t ^2)$. In view of that scheme the system (17) can be discretized as

38 $$\begin{aligned} \begin{aligned} S_{j+1}&=S_j+\Big (-\iota _a S_j+\iota _g C_j\Big )\Delta t-\sigma _1 S_j \sqrt{\Delta t}\tau _j-\frac{\sigma _1^2}{2}S_j(\tau ^2_j-1)\Delta t,\\ R_{j+1}&=R_j+\Big (\iota _anS_j-\iota _rR_j-\iota _b R_jM_j\Big )\Delta t+\sigma _1 n S_j \sqrt{\Delta t}\tau _j+\frac{\sigma _1^2n^2}{2}S_j(\tau ^2_j-1)\Delta t\\&-\sigma _2R_j\sqrt{\Delta t}\tau _j-\frac{\sigma _2^2}{2}R_j(\tau ^2_j-1)\Delta t\\ C_{j+1}&=C_j+\Big (\iota _b R_jM_j-(\iota _g+\iota _c)C_j\Big )\Delta t-\sigma _3 C_j\sqrt{\Delta t}\tau _j-\frac{\sigma _3^2}{2}C_j(\tau ^2_j-1)\Delta t, \\ M_{j+1}&=M_j+\Big (\iota _h-\iota _mM_j-\iota _bR_jM_j\Big )\Delta t-\sigma _4 M_j\sqrt{\Delta t}\tau _j-\frac{\sigma _4^2}{2}M_j(\tau ^2_j-1)\Delta t, \end{aligned} \end{aligned}$$

where the value of the ${i}^{{th}}$ iteration of the discretization equation is denoted by $(S_j, R_j,C_j,M_j)$, the time increment is denoted by $\Delta t> 0$ and $\tau ^2_j$ $(j = 1, 2,...)$ represents the independent random variables with a Gaussian distribution ${\mathcal {N}}(0,1)$. The deterministic counter part with zero noise (i.e., $\sigma _1=\sigma _2=\sigma _3=\sigma _4=0$) of the discretized system (38) results in the Euler scheme for model (1) that is

39 $$\begin{aligned} \begin{aligned} S_{j+1}&=S_j+\Big (-\iota _a S_j+\iota _g C_j\Big )\Delta t,\\ R_{j+1}&=R_j+\Big (\iota _anS_j-\iota _rR_j-\iota _b R_jM_j\Big )\Delta t,\\ C_{j+1}&=C_j+\Big (\iota _b R_jM_j-(\iota _g+\iota _c)C_j\Big )\Delta t, \\ M_{j+1}&=M_j+\Big (\iota _h-\iota _mM_j-\iota _bR_jM_j\Big )\Delta t. \end{aligned} \end{aligned}$$

**Fig. 1:**
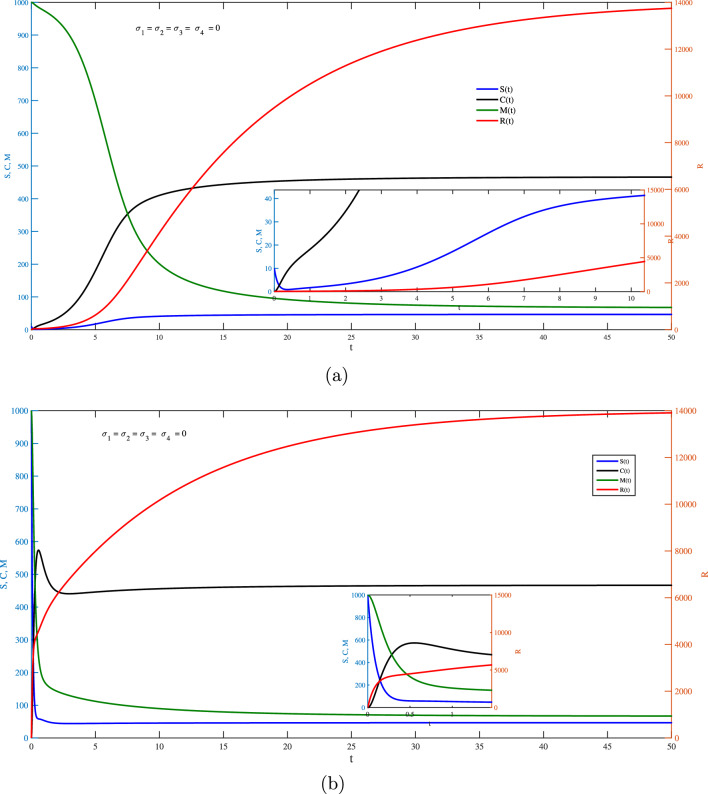
Influence of *S*, *R*, *C* *and* *M* via *t* for the deterministic model (1) with (**a**) $S(0)=10, { and ({\textbf {b}}) } S(0)=1000$.

To this end, Fig. 1 represents the influence of *S*, *R*, *C* *andM* via *t* for the deterministic model (1) with the values of the parameters of the model as well as the initial values of the variables given in Table 1 with (a) $S(0)=10, { and (b) } S(0)=1000$. In the plots, the values of *S*, *C* *andM* are shown in the left vertical axis, while the values of *R* are shown in the right vertical axis due to the significant difference between the two sets of values. In Fig. 1a, the concentration of dsRNA $(S(0)=10)$ (low value), that is required at all cases to be non-zero for the dynamics to be evolved, started to decrease slightly along the starting time evolution and increases again until it reaches a steady state. Further, the silencing reaction can take off and eventually attain a new steady state with significantly decreased amounts of mRNA and a continual silencing reaction. In other words, once the silencing reaction is activated, it can strengthen itself and establish a new steady state with significantly lower mRNA levels. This behavior can be attributed to positive feedback loops in the RNA silencing pathway. Once the silencing reaction is initiated, mechanisms such as RISC and RNA-dependent RNA polymerases help develop the silencing effect by generating more siRNAs or miRNAs. This leads to continuous degradation of the target mRNA, reinforcing the silencing state and establishing a new steady state with significantly lower mRNA levels. For the high concentration of dsRNA $(S(0)=1000)$ Fig. 1b is presented in which one can find that the concentration of both dsRNA and mRNA decreases with the starting time until the steady state is achieved. A key point here is that any initial dose of dsRNA (low or high) will result in the same final degree of silence.

**Fig. 2:**
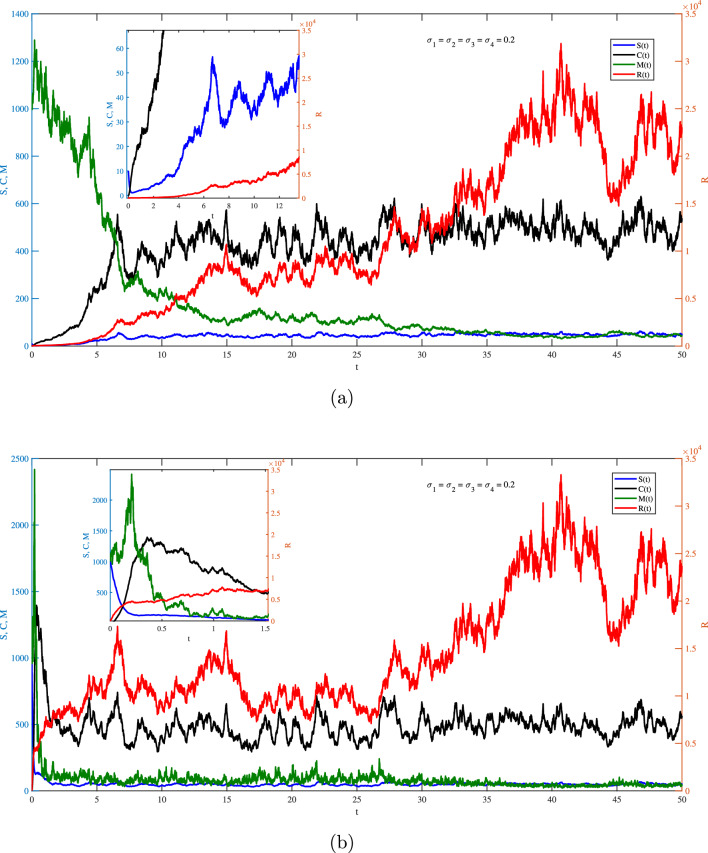
Stochastic fluctuations of *S*, *R*, *C* *and M* via *t* from the model (17) at $\sigma _i=0.2, i =1,2,\cdots , 4$ with (**a**) $S(0)=10, { and ({\textbf {b}}) } S(0)=1000$.

**Fig. 3:**
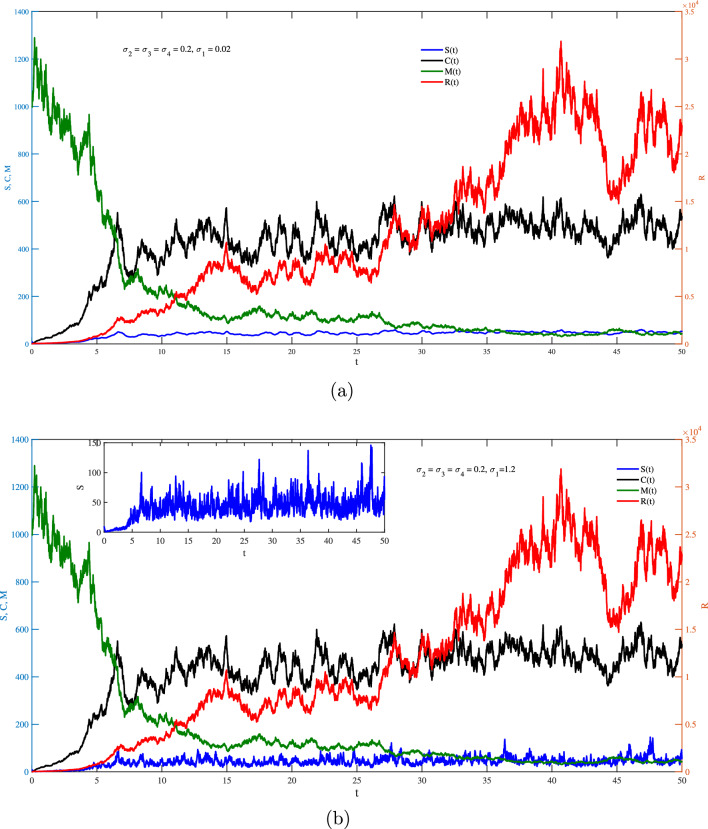
Stochastic fluctuations of *S*, *R*, *C* *and M* via *t* from the model (17) with $S(0)=10$ for (**a**) $\sigma _1=0.02,$ and (**b**) $\sigma _1=1.2$.

**Fig. 4:**
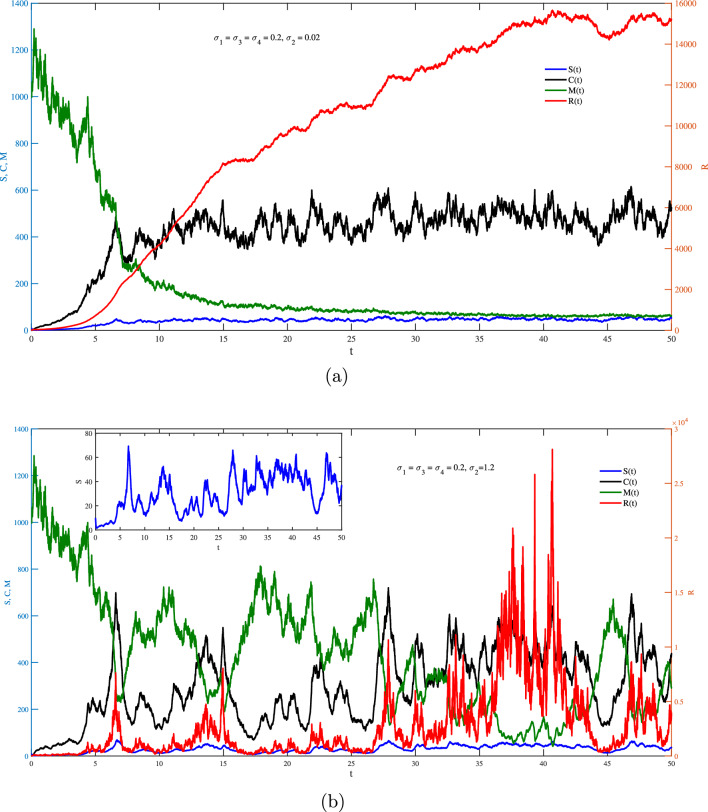
Stochastic fluctuations of *S*, *R*, *C* *and M* via *t* from the model (17) with $S(0)=10$ for (**a**) $\sigma _2=0.02,$ and (**b**) $\sigma _2=1.2$.

**Fig. 5:**
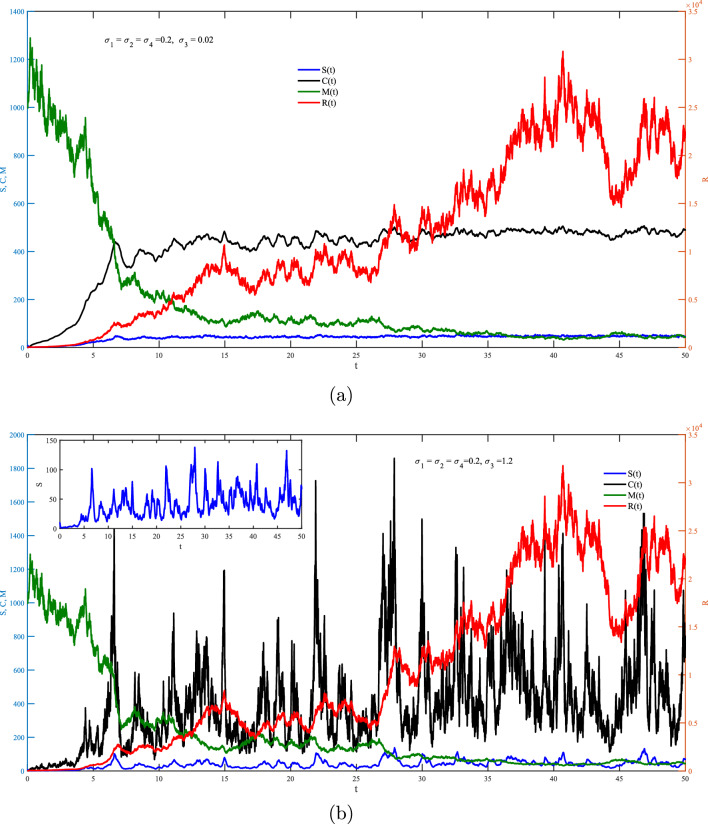
Stochastic fluctuations of *S*, *R*, *C* *and M* via *t* from the model (17) with $S(0)=10$ for (**a**) $\sigma _3=0.02,$ and (**b**) $\sigma _3=1.2$.

**Fig. 6:**
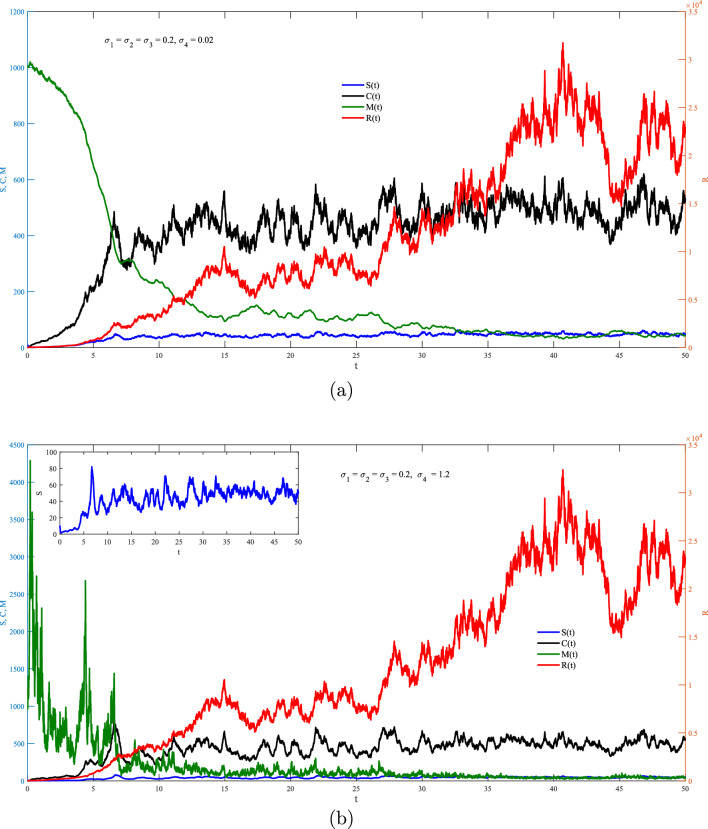
Stochastic fluctuations of *S*, *R*, *C* *and M* via *t* from the model (17) with $S(0)=10$ for (**a**) $\sigma _4=0.02,$ and (**b**) $\sigma _4=1.2$.

One the other hand, Figs. 2–6 show the case of adding random effects to the model and observing certain safety procedures, these simulations aid in our investigation of the dynamical behaviors of the model. Fig. 2 shows the trajectories of solution for the stochastic model (17) for $\sigma _1 = \sigma _2 =\sigma _3=\sigma _4=0.2$ with (a) $S(0)=10$ (low initial concentration of dsRNA) and (b) $S(0)=1000$ (high initial concentration of dsRNA). In the stochastic model, various doses for the concentration of dsRNA show a similar overall trend in the evolution of *S*(*t*) as observed in the deterministic model. However, the stochastic model also accounts for random fluctuations in the variables due to inherent noise in the system, unlike the smooth trajectory seen in the deterministic case. This fluctuation is attributed to the involvement of the environmental effects represented by the white noise. Hence, this recommends that while the general behavior is maintained across models, the stochastic model better captures biological adaptability. Integrating noise into the coefficient of dsRNA in an RNA silencing model (17) reflects model reflects the inherent biological variability in the production, processing, and degradation of dsRNA molecules. Factors such as fluctuating enzyme activity, varying cellular environments, viral interference, and external stressors can all result in unpredictable changes in dsRNA dynamics. By treating this coefficient as a stochastic parameter, the model captures these random fluctuations, offering a more realistic representation of the RNA silencing process. This tackle improves the model’s ability to replicate the robustness and adaptability of gene regulation mechanisms under naturally noisy biological conditions. To this end, impacts of $\sigma _1$ on different variables of the model is presented in Fig. 3a and b. It is clear significant different fluctuations on *S*(*t*) are obtained for the case of low value $(\sigma _1=0.02)$ compare to the high value $(\sigma _1=1.2)$. The other variables of the model *R*(*t*), *C*(*t*),  and *M*(*t*) does not significantly affected by increasing the values of $\sigma _1$. Noting that both values of $\sigma _1$ are in the stable region of the model. Fig. 4 explores the effects of $\sigma _2$ on *S*(*t*), *R*(*t*), *C*(*t*) and *M*(*t*) with (a) $\sigma _2=0.02$ and (b) $\sigma _2=1.2$. It is very obvious from the plots that changing $\sigma _2$ affects directly the fluctuation of *R*(*t*), and also it has a very significant impacts on the fluctuations of *C*(*t*) and *M*(*t*) . This effect is mathematically attributed to the term $\iota _{b} R(t)M(t)$ that exists in the last three equation of the model (17). Note that incorporating a stochastic coefficient for RISC in the RNA silencing model captures the inherent variability in its formation and activity caused by fluctuating protein levels, molecular interactions, and environmental stress, thereby enhancing the model’s biological accuracy and offering deeper insight into the robustness of gene silencing under dynamic cellular conditions.

Further, significant changes on the fluctuation of *C*(*t*) is obtained when changing $\sigma _3$ from 0.02 to 1.2 as shown in Fig. 5. A similar behavior is obtained for the fluctuation of *M*(*t*) as $\sigma _4$ goes from 0.02 to 1.2 in Fig. 6. One may note that comparing the fluctuations of *R*(*t*) and *C*(*t*) between Fig. 6a and b, there is a shift down of the fluctuation of *C*(*t*). This is just because of the change of the scale of the left vertical axis while the right one remains invariant. From all the simulation, Nevertheless, the stochastic or deterministic scenario, all simulations confirms that the solution is positive. Also, the key conclusion point in the deterministic case, that is beginning with any dose of dsRNA (low or high) will led to the same final level of silence, is reported for the stochastic scenario. In the obtained simulation, values of the stochastic parameters have been chosen to verify the conditions (37) and as a result the system is globally asymptotically stable. Finally, the stochastic stability of the RNA model authorizes that the system stays well-behaved under random environmental fluctuations. This not only supports the model’s biological relevance but also provides a solid foundation for future applications in gene regulation, synthetic biology, and RNA-based therapeutics.

## Conclusion

This research explores the impact of stochastic environmental fluctuations on the RNA silencing model leading to a novel stochastic RNA silencing model. Stability analysis is conducted for both the stochastic model and its deterministic counterpart, allowing for a comprehensive understanding of system behavior under different conditions. The proposed stochastic model admits a globally asymptotically stable point. Existence and uniqueness of a positive global solution for the proposed model is ensured. Accurate numerical simulation based on the Milstein’s higher order method is presented in order to ensure the theoretical results. All simulations of the stochastic or deterministic scenario confirm that the solution is positive. Starting with any dose of dsRNA (low or high) leads to the same final level of silence. Key point of the finding is that, considering random effects for the parameters of the model reflects a more realistic aspect of the development of the biological phenomena and offering deeper insight into the robustness of gene silencing under dynamic cellular conditions. As a future work, the model could be extended to merge spatial dynamics, as diffusion and intracellular localization of RNA molecules and silencing components, which would allow investigation into how spatial heterogeneity within the cell influences the efficiency and regulation of gene silencing. Furthermore, considering the simulation of the model based on artificial intelligence is of interest as well.

## Data Availability

All the used data are included in the article.
